# Iron overload induced by ferric derisomaltose and ferric carboxymaltose both increase FGF-23 levels and lead to osteomalacia and bone loss in normal mice

**DOI:** 10.1007/s10534-026-00794-x

**Published:** 2026-02-13

**Authors:** Xuan-Thanh Le-Phuoc, Vanessa Passin, Maria G. Ledesma-Colunga, Heike Weidner, Imke Fiedler, Björn Busse, Ulrike Baschant, Lorenz C. Hofbauer, Martina Rauner

**Affiliations:** 1 Department of Medicine III Center for Healthy Aging, Medical Faculty, University Hospital Carl Gustav Carus, Dresden University of Technology, Fetscherstr. 74, 01307 Dresden, Germany; 2 Department of Osteology and Biomechanics, University Medical Center Hamburg-Eppendorf, Hamburg, Germany; 3 Interdisciplinary Competence Center for Interface Research (ICCIR), University Medical Center Hamburg-Eppendorf (UKE) Hamburg, Hamburg, Germany

**Keywords:** Intravenous iron, Osteomalacia, Bone mineralization, Fibroblast growth factor (FGF)-23

## Abstract

Ferric carboxymaltose (FCM) and ferric derisomaltose (FDI) are key for treating iron deficiency anemia. However, FCM has been shown to raise serum fibroblast growth factor (FGF)-23 levels, causing hypophosphatemia and alterations in bone turnover in some patients. To date, it is unknown if FCM and FDI also affect bone mineralization. This study examined FDI and FCM effects on bone mineralization and FGF-23 in healthy mice, avoiding disease confounders. Male 12-week-old C57BL/6 J mice received single or weekly FDI, FCM, or placebo injections for 4 weeks.Repeated FDI and FCM injections affected body weight, blood counts, and caused significant liver iron accumulation and high serum iron. Both reduced most bone parameters by µCT; however, FCM showed falsely high bone density due to iron clusters in the bone marrow. Histology revealed greater bone volume loss with FCM than FDI (-36% FCM, *p* < 0.01; vs − 24% FDI, *p* < 0.05), predominantly from suppressed bone formation. Both iron formulations also led to a prominent increase in osteoid and FGF-23 (intact and C-terminal), raising the i:cFGF-23 ratio. A single dose led to similar, but milder effects than repeated dosing. In summary, repeated high doses of both, FDI and FCM, in healthy mice increased the i:cFGF-23 ratio and osteoid production, while reducing bone formation and volume.

**Supplementary Information:**

The online version contains supplementary material available at 10.1007/s10534-026-00794-x.

## Introduction

Iron is essential for several physiological processes, including oxygen transport via hemoglobin (Perutz et al. 1998; Ponka 1999) and myoglobin (Ponka 1999; Ordway and Garry 2004), energy production in mitochondria (Rouault and Tong 2008; Sheftel et al. 2012; Lill et al. 2012) and for the activity of enzymes involved in DNA replication and the cell cycle (Cazzola and Skoda 2000; Puig et al. 2017). As such, well-balanced iron levels are indispensable for health, with iron deficiency causing health problems such as anemia, weakness (Lopez et al. 2016) and shortness of breath. Because of its importance in various biochemical processes, the causes of iron deficiency are diverse, ranging from increased physiological demand, such as menstrual blood loss or pregnancy, to inadequate intake due to poverty, malnutrition or diet preference. Iron deficiency can also be a result of genetic diseases like iron-refractory iron-deficiency anemia or other pathologies that disturb the ability of iron absorption, such as bacterial or parasite infections, celiac disease, and chronic inflammatory diseases. To treat iron-deficiency anemia, oral iron therapy is usually sufficient. However, oral iron supplementation can cause gastrointestinal side effects (Pantopoulos 2024) and worsen the conditions (Erichsen et al. 2005; Kortman et al. 2014; Camaschella 2015) in the latter groups of patients and therefore needs to be replaced by parenteral therapy.

Indeed, different formulations for intravenous administration of iron have been developed to overcome the gastrointestinal adverse effects of the oral therapy. The formulations have been further improved over time to increase the amount of iron administration in each infusion while effectively preventing the toxicity of free labile iron in circulation. The introduction of ferric carboxymaltose (FCM) made it possible to administer 1000 mg of iron within a single dose. Ferric derisomaltose (FDI) is the most recent formulation that requires no test dose and can be administered up to 20 mg/kg body weight. Both of them have been shown to be efficacious in resolving iron-deficiency anemia and are better tolerated than oral iron therapy. Recommended by the European Crohn's and Colitis Organization as a first-line treatment, FCM and FDI became the most widely used treatments for iron-deficiency anemia in Europe.

Despite the rapid correction of iron-deficiency anemia, both formulations come with potential side effects. FDI is frequently associated with hypersensitivity reactions, which require immediate medical attention, while FCM has been reported to induce transient hypophosphatemia in many patients. The induction of hypophosphatemia has been associated with impaired tubular phosphate resorption and low levels of serum 1,25-(OH)_2_D_3_, mimicking hypophosphatemic conditions caused by excess actions of fibroblast growth factor-23 (FGF-23), such as X-linked hypophosphatemia or tumor-induced osteomalacia (Shimizu et al. 2009). Even though most cases of FCM-induced hypophosphatemia appear to be transient, severe cases of iron-deficiency anemia may require multiple doses of iron to correct red blood cell levels. Thus, it may be possible that repeated doses of intravenous iron could lead to prolonged hypophosphatemia, which may cause osteomalacia, characterized by impaired bone mineralization, pain, and fragility (Zoller et al. 2017; Bartko et al. 2018; Struppe et al. 2023). Case reports have revealed bone pain and osteomalacia after prolonged treatment with FCM (Boots and Quax 2022). Overall, the mechanisms underlying iron-induced FGF-23 levels and hypophosphatemia are not well understood.

In this study, we administered high doses of FDI and FCM parentally to healthy adult male mice to better understand the effects of iron on FGF-23 levels and bone mineralization excluding confounders from other underlying diseases. We used single-dose and multiple-dose applications to mimic different clinical scenarios of short-term vs. long-term treatment with iron. Besides the expected iron overload in liver, serum and bone marrow, we show that both iron formulations were associated with bone loss due to severely impaired bone formation. Importantly, both iron formulations elevated the ratio of intact to C-terminal FGF23 (i:cFGF23) and the amount of osteoid. As expected, repeated doses of FDI and FCM produced stronger effects than a single-dose application, suggesting that for bone health, as few iron injections as possible should be used.

## Materials and methods

### Iron application in vivo

Twelve-week-old male and female C57BL/6 J mice received 0.5 g/kg body weight iron (either ferric derisomaltose (FDI, Monofer), Pharmacosmos, ferric carboxymaltose (FCM, Ferinject), CSL Vifor, or iron dextran (ID), Sigma, intraperitoneal injections) per week for four weeks according to previous publications(Daba et al. 2013; Robin et al. 2023). In another set of experiments, FDI or FCM was injected once at a dose of 0.5 g/kg body weight and mice were sacrificed 4 weeks later. Mice were fed a standard rodent diet (198 ppm iron) with water ad libitum and were held under a 12 h light/dark cycle and in an air-conditioned room at 23 °C. Weight was monitored every week. Mice were euthanized at the age of 16 weeks under deep anesthesia and blood, organs and bones were collected for further analysis. Animal procedures were approved and conducted in compliance with the guidelines of the institutional animal care committee and the Landesdirektion Sachsen (TVV 20/2020).

### Blood counts

Blood counts were measured in the peripheral blood of the mice. At sacrifice, blood was collected via heart puncture, diluted with PBS, and analyzed with a Sysmex XN-1000.

### Iron measurements

Non-heme iron content in the collected liver and serum was measured using the bathophenanthroline colorimetric method (SFBC) as previously described (Torrance and Bothwell 1968; Rauner et al. 2019). Briefly, 100 mg of liver tissue was dried for three days at 45 °C and afterwards the samples were incubated with 0.01% bathophenanthrolinedisulfonic acid. Values were recorded spectrophotometrically at 535/540 nm. Non-heme iron content is reported as µg iron/g dry tissue weight.

Iron content in histological sections was performed using Perl’s Prussian Blue staining as previously published (Dogan et al. 2024). Iron-loaded cells were quantified in the femur in an area of 0.24 mm^2^. Iron clusters were identified as larger conglomerates of cells stained with iron and were marked as an area. Iron-covered bone surface was quantified as well using the Osteomeasure software (Osteometrics, USA).

### µCT analysis of bone microarchitecture

Bones were measured ex vivo at the end of the experiment. The distal femur and the fourth lumbar vertebra were excised and scanned using a resolution of 10.5 µm with a vivaCT40 (Scanco Medical, Switzerland). For the femora, half the femur was scanned in the scout view of which 100 slices below the growth plate of the distal femur were evaluated for trabecular bone, and 150 slices in the mid-diaphysis were evaluated for cortical bone. For analysis of bone volume, the same slices as for cortical bone were taken, but the contours included the bone marrow space instead of the cortical bone. For the vertebral bone, the entire 4th lumbar vertebra was scanned and 100 slices in the middle of the bone were measured. Trabecular and cortical bone parameters were assessed using standard protocols from Scanco Medical. µCT parameters are reported according to international guidelines(Bouxsein et al. 2010; Dempster et al. 2013).

### Biomechanical testing

A three-point bending flexural test of the femoral diaphysis was performed to assess bone strength. The femora were stored in 70% ethanol and rehydrated in PBS for 24 h before testing. A Zwick/Roell machine type Z2.5 from Zwick, Germany was used to conduct the mechanical test. Mechanical load was applied to the anterior side of the femoral shaft to measure the maximum load at failure (Fmax, N) *and the elastic modulus (Emod, MPa)*.

### Quantitative backscattered electron imaging (qBEI)

Embedded sample blocks were ground and fine-polished and their co-planar surface was sputtered with carbon. Using 20 kV voltage and constant working distance, backscattered-electron images of the vertebral bone were acquired at a magnification of 150 × using an electron microscope (Zeiss Crossbeam 340). Images were calibrated based on standards of carbon and aluminum according to previously established protocols, and the conversion of gray values to calcium wt % was performed using a custom-written Matlab script. Five mineralization density distribution parameters including the weighted mean calcium-concentration of the bone area (Ca Mean), the peak position of the histogram (Ca Peak), the percentage of highly mineralized bone areas (Ca High), the percentage of lowly mineralized bone areas (Ca Low), and CaWidth (assessed as the full width at half maximum (FWHM) of the histogram curve) as measure for heterogeneity of mineral concentrations were determined as previously described (Roschger et al. 1998, 2008; Milovanovic et al. 2015).

### Bone histomorphometry

All mice received two intraperitoneal injections with 20 mg/kg calcein (Sigma) five and two days before sacrifice. For dynamic bone histomorphometry, the third and fourth lumbar vertebrae were fixed in 4% PBS-buffered paraformaldehyde and dehydrated in an ascending ethanol series. Subsequently, bones were embedded in methacrylate and cut into 7 µm sections to assess the fluorescent calcein labels. Sections were analyzed using fluorescence microscopy to determine the mineralized surface/bone surface (MS/BS), the mineral apposition rate (MAR), and the bone formation rate/bone surface (BFR/BS). To assess the osteoid volume (OV), surface (OS) and width (O.Wi), 4 μm methacrylate sections were stained with von Kossa/van Gieson. The Osteomeasure software (Osteometrics, USA) was used to analyze an area of 1.44 mm^2^.

To determine surface area and numbers per bone perimeter of osteoblasts as well as osteoclasts (Ob.S/BS, N.Ob/B.Pm, Oc.S/BS, N.Oc/B.Pm, respectively), the fifth lumbar vertebra was decalcified for one week using Osteosoft (Merck), dehydrated, and embedded into paraffin. Two-micron paraffin sections were then stained with tartrate-resistant acid phosphatase (TRAP). Again, an area of 1.44 mm^2^ was analyzed using the Osteomeasure software. Pictures were taken using the CellSens program while fluorescence pictures were taken using the AxioVision 4.8 program.

### Serum analysis of bone turnover markers

Serum concentrations of pro-collagen type I amino-terminal propeptide (P1NP), C-terminal telopeptide of type I collagen (CTX), and tartrate-resistant acid phosphatase 5b (TRAP5b) were quantified using ELISAs from Immundiagnostik, Bensheim, Germany. Serum levels of C-terminal and intact FGF-23 were also measured with ELISAs from QuidelOrtho, USA, and serum phosphate was quantified using a Phosphate Assay Kit from Abcam, Cambridge, UK.

### Quantitative real-time polymerase chain reaction (qRT-PCR)

The extraction of total RNA from bone marrow and flushed bone tissue was performed separately using TRIzol reagent (Invitrogen). The RNA was reverse-transcribed using random primers (Thermo Fisher Scientific), dNTPs (Roth), M-MLV Reverse Transcriptase and RNasin (both from Promega). The gene expression was then measured by qRT-PCR (QuantStudio 5, Applied Biosystems) with PowerUp SYBR Green Master Mix (Thermo Fisher Scientific). The relative expression was calculated by 2^−ΔΔCt^ method and normalized to the reference gene *Actb* (*beta-actin)*. Primers used in this investigation were purchased from Sigma-Aldrich (Thermo Fisher Scientific) with the following sequences: *Galnt3*: Forward: 5’-TCTTCACCTGCCATACTGCTG-3’, Reverse: 5’-TTCCTTTCTTGCTGCCTGAC-3’; *Fam20C*: Forward: 5’- ACATGACCAAGGAGATCCG-3’, Reverse: 5’- CAGATGTTGTTGGCTGGAG-3’; *Furin*: Forward: 5’- ACAACTATGGGACGCTGAC-3’, Reverse: 5’-TGCTTTCTGGAGGTGTAGAG-3’; *Actb*: Forward: 5’- GATCTGGCACCACACCTTCT-3’, Reverse: 5’- GGGGTGTTGAAGGTCTCAAA-3’.

### Statistical analysis

Data are presented as mean ± standard deviation (SD) with presentation of individual data points. Comparisons among three groups (FDI vs. FCM vs. PBS) were performed using one-way ANOVA followed by Tukey’s post-hoc multiple comparison tests. Comparisons between two groups (iron dextran vs. PBS) were conducted using two-sided Student’s *t*-tests. All statistical analysis were performed using GraphPad Prism 10 (GraphPad Software Inc, USA). A p-value of < 0.05 was considered statistically significant.

## Results

### Mice treated with FDI or FCM become iron overloaded

**Fig. 1:**
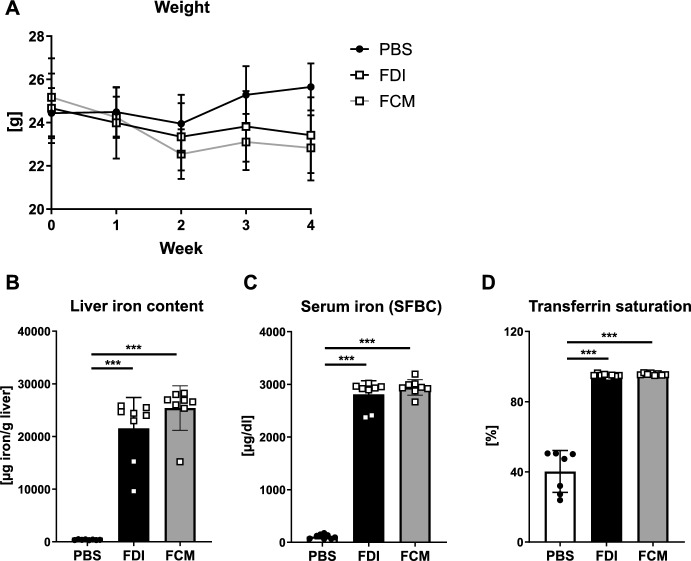
Iron overload induced by FDI and FCM treatments in mice. Twelve-week old male C75BL/6 J mice received 0.5 g iron/kg body weight per week for four weeks (either FDI or FCM). **A** Body weight, **B** liver iron content, **C** serum iron levels (SFBC), and **D** serum transferrin saturation were assessed. Individual dots represent individual mice. Mean and SD are indicated as horizontal lines. Eight mice per group were used. A one-way ANOVA was used for statistical analysis. ***p < 0.001

To investigate how our treatment scheme with FDI or FCM affects the general health of mice, we monitored their body weight weekly and analyzed their blood counts as well as their liver iron content at the end of the experiment. Both FDI and FCM led to a reduction of body weight towards the end of the experiment (Fig. 1A, FDI: − 9%, *p* < 0.01; FCM: − 12%, *p* < 0.001). Moreover, assessing the liver iron content revealed a heavy iron deposition in the liver with both iron formulations (Fig. 1B). This was accompanied by high serum iron levels and iron-saturated transferrin levels, reaching nearly 100% (Fig. 1C–D). Applying another iron formulation, iron dextran (ID), at the same dose to mice resulted in similar effects, with the mice showing a 5% decrease in body weight (*p* < 0.05) and a high amount of iron in the liver (Suppl. Figure 1A, B). To account for skeletal sexual dimorphism, we broadened our examination to female mice receiving repeated FCM and FDI injections. The results mirrored those observed in males: both iron formulations increased hepatic and serum iron content and elevated transferrin saturation levels, confirming systemic iron overload (Suppl. Figure 2A–C).

Concerning the blood counts, FCM and ID overall showed a similar profile, while FDI showed milder effects (Table 1). FCM and ID significantly reduced the red blood cell count as well as the hematocrit and hemoglobin (Table 1). Mean corpuscular volume was reduced by all three iron treatments (Table 1). All iron sources led to an increase in white blood cells, with FCM and ID showing the largest increase (Table 1). In particular, the numbers of monocytes and neutrophils were increased, while lymphocytes were decreased in number (Table 1). In contrast, these effects were absent in female mice treated with FDI and FCM (Suppl. Table 2). Finally, all treatments led to a reduction in reticulocytes in males and females (Table 1 and Suppl. Table 2). Thus, all iron treatments led to a comparable iron overload and inflammatory profile after 4 weeks of treatment in male mice, while female mice showed a favorable outcome regarding blood cell parameters.

**Table 1:** Blood counts in mice treated with multiple doses of iron

	Control *N* = 8	FDI *N* = 8	FCM *N* = 8	Control *N* = 8	Iron dextran *N* = 8
Red blood cells [10^6^/ µl]	9.95 ± 0.75	9.38 ± 0.44	8.78 ± 0.49**	9.59 ± 0.34	8.33 ± 1.11*
Hematocrit [%]	48.7 ± 3.84	44.6 ± 2.72*	41.6 ± 2.58***	48.0 ± 2.00	40.0 ± 5.71**
Hemoglobin [g/dl]	9.08 ± 0.63	8.63 ± 0.56	8.14 ± 0.46**	8.85 ± 0.42	7.69 ± 1.01*
MCV [fl]	48.95 ± 0.61	47.43 ± 0.64***	47.38 ± 1.23**	49.98 ± 0.90	48.28 ± 0.67**
MCH [pg]	0.91 ± 0.025	0.92 ± 0.021	0.93 ± 0.024	0.92 ± 0.02	0.92 ± 0.01
MCHC [g/dl]	18.64 ± 0.43	19.33 ± 0.41**	19.59 ± 0.57**	18.48 ± 0.48	19.13 ± 0.24**
Platelets [10^3^/ µl]	317.8 ± 29.4	278.0 ± 31.0*	225.6 ± 25.6***	327.0 ± 191.2	648.4 ± 109.4
White blood cells [10^3^/µl]	10.86 ± 2.39	14.68 ± 3.70*	17.95 ± 3.80***	9.42 ± 4.05	18.23 ± 5.32**
Neutrophils [%]	8.99 ± 1.44	12.38 ± 4.13	14.8 ± 3.80**	5.80 ± 1.77	7.99 ± 2.86
Lymphocytes [%]	87.63 ± 2.18	72.09 ± 14.16*	66.34 ± 6.55***	91.79 ± 2.69	85.21 ± 7.59*
Monocytes [%]	2.86 ± 1.26	15.08 ± 11.97*	18.21 ± 6.67***	2.16 ± 1.73	4.14 ± 1.57*
Reticulocytes [10^9^/L]	232.4 ± 23.97	130.2 ± 37.4***	113.3 ± 75.34***	236.8 ± 48.85	91.76 ± 18.15***

*MCV* mean corpuscular volume, *MCH* mean corpuscular hemoglobin. *MCHC* mean corpuscular hemoglobin concentration

Data represent the mean ± SD. Statistical analysis was conducted using the Student´s *t*-test for comparisons between control and iron dextran, and one-way analysis of variance (ANOVA) followed by Tukey’s post-hoc test for comparisons among Control, FDI and FCM. *p < 0.05; **p < 0.01; ***p < 0.001

### FDI and FCM show distinct bone and bone marrow characteristics

**Fig. 2:**
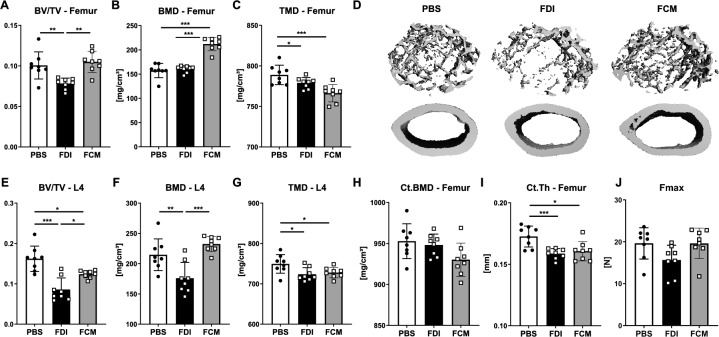
Bone microarchitecture and bone strength of FDI- and FCM-treated mice. **A** Fraction of bone volume over total volume (BV/TV), **B** bone mineral density (BMD), and **C** tissue mineral density (TMD) of the distal femur were assessed using µCT. **D** 3D reconstructions represent the morphology of trabecular and cortical bone of the femur. **E** BV/TV. **F** BMD, and **G** TMD of the fourth vertebral body. **H** Cortical BMD and **I** cortical thickness (Ct.Th) assessed at the mid-shaft using µCT. **J** Maximal load at failure (Fmax) was measured by three-point bending of the femur. Individual dots represent individual mice. Mean and SD are indicated as horizontal lines. Eight mice per group were used. A one-way ANOVA was used for statistical analysis. *p < 0.05, **p < 0.01, ***p < 0.001

Next, we investigated the bone microarchitecture of the mice using µCT and tested their bone strength using biomechanical tests (Fig. 2). Except for bone mineral density (BMD) at the distal femur, FDI led to the expected reductions of trabecular bone volume, BMD and tissue mineral density (TMD) at the femur and fourth vertebral body (Fig. 2A–G). Moreover, FDI did not alter the BMD at the femoral cortical bone, but led to a reduction in cortical thickness (Fig. 2H–I). This phenotype resulted in a reduction of bone strength (Fig. 2J). Interestingly, FCM led to different outcomes, showing only a reduction of bone volume at the spine, but no alterations in volume at the femur and even increased (femur) or a trend to an increased (spine) trabecular BMD, which is also reflected by the representative image of the bone microarchitecture (Fig. 2A, B, D, E, F). Importantly, TMD was decreased by FCM treatment at both sites (Fig. 2C, G). Cortical bone was negatively affected by FCM treatment, showing reduced BMD and thickness of the cortex (Fig. 2H, I). Female mice showed a similar pattern (Suppl. Figure 2D–L). ID treatment did not lead to any major alterations in trabecular or cortical bone volume, BMD or TMD (data not shown) of the spine or femur (Suppl. Figure 1C–G).

**Fig. 3:**
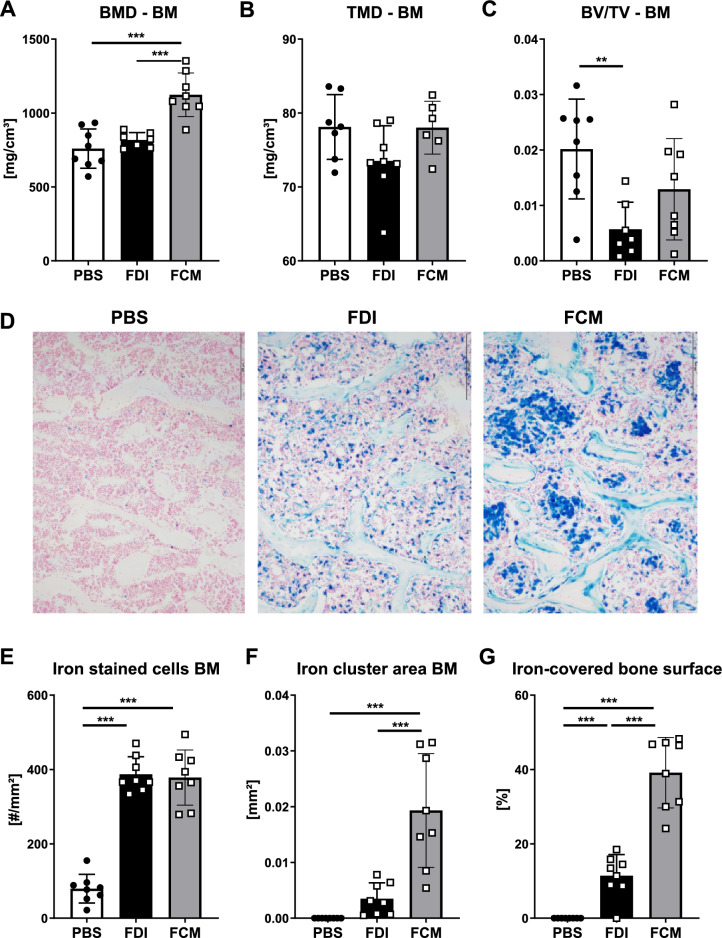
Analysis of iron deposition in the bone marrow. **A** Bone mineral density (BMD), **B** tissue mineral density (TMD), **C** Bone volume fraction (BV/TV) of the bone marrow compartment in the mid-femur was assessed using µCT. **D** Representative staining of iron in the bone marrow compartment (blue dots indicate iron deposition), scale bar: 200 µm. **E-G** Iron deposition in the bone marrow was quantified via histology. **E** Number of iron-stained cells, **F** iron cluster area, and **G** iron-covered bone surface. Individual dots represent individual mice. Mean and SD are indicated as horizontal lines. Eight mice per group were used. A one-way ANOVA was used for statistical analysis. **p < 0.01, ***p < 0.001

As TMD was decreased in the FCM-treated group, which only takes the mineralized tissue into account, but not BMD, which considers both the mineralized and soft tissues, we wondered if some of the “density” signal could stem from iron deposition in the bone marrow. To that end, we evaluated the bone marrow compartment of the femoral midshaft using the same BMD threshold as for the trabecular bone analysis of the distal femur. Indeed, mice treated with FCM showed a higher BMD in the bone marrow compartment as compared to PBS or FDI-treated mice (Fig. 3A). Bone volume and TMD, which in general were very low in the bone marrow compartment, were decreased in the FDI group, but not in the FCM group (Fig. 3B–C). Histological analyses of the bones confirmed the µCT data, showing a significant accumulation of iron in the bone marrow of FDI- and FCM-treated mice, however, with distinct forms of deposition (Fig. 3D). While FDI resulted in iron-loaded macrophages scattered throughout the bone marrow, FCM led to a significant accumulation of iron in “iron clusters”, which may result in the increased BMD signal in the µCT (Fig. 3E–F). Both iron treatments increased the amount of iron-covered surface, but FCM showed the largest increase (Fig. 3G). ID resulted in a similar pattern of iron deposition in the bone marrow as FDI (Suppl. Figure 1H).

Taken together, both FDI and FCM reduced bone TMD, but only FDI also led to reductions in trabecular bone volume and BMD. This may stem from the major accumulation of iron clusters in the bone marrow of FCM-treated mice, which may provide a “false-positive” signal for BMD measurements using µCT.

### Bone mineral density distribution is not affected by FDI or FCM

**Fig. 4:**
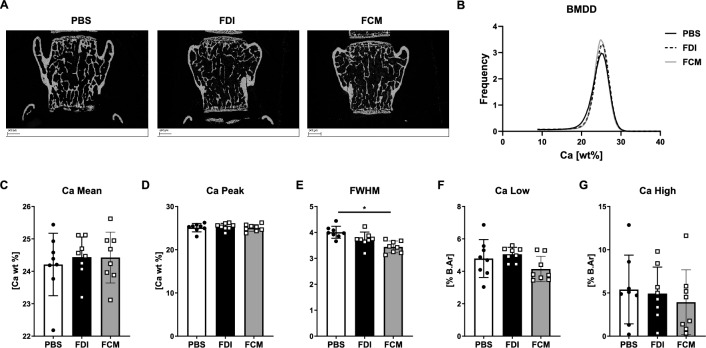
Bone mineral density distribution in FDI- and FCM-treated mice. **A** Frontal sections of vertebral bodies were analyzed **B** The histogram of calcium concentration indicates a similar bone mineral density distribution with narrower curves in treated mice **C** Mean calcium concentration (Ca Mean) and **D** peak calcium concentration (Ca Peak) were similar in all groups. **E** Full width at half maximum (FWHM) of the absorption peaks, a measure of mineralization heterogeneity was significantly lower in FCM-treated mice**. F** Areas of low and high mineralization (Ca Low, Ca High, respectively) were similar between the groups, while a trend towards fewer low and fewer high mineralized areas was visible in FCM-treated mice, supporting a narrower, more homogenous calcium concentration. ANOVA with Tukey’s post hoc tests was performed to compare the groups. *p < 0.05

We further analyzed the bone mineral density distribution (BMDD) in the mature trabecular bone region of vertebral bodies of mice treated with PBS, FDI and FCM (Fig. 4A–B), which only showed minor differences between the groups. Mean and peak calcium concentrations were similar between all groups (Fig. 4C, D). CaWidth (assessed as FWHM), a measure for the heterogeneity of the calcium concentration, showed a trend towards narrower curves in mice treated with FDI (*p* = 0.08) and a significantly lower value in FCM-treated mice (*p* < 0.001) (Fig. 4E). CaLow and CaHigh, measures indicating percentage areas with low and high calcium concentrations, respectively, did not show clear differences between the groups. However, in FCM-treated mice, slight trends towards lower bone areas with high and low mineralization indicate a more homogeneous calcium concentration within vertebral bone (Fig. 4F–G).

### FDI and FCM significantly reduce bone formation

**Fig. 5:**
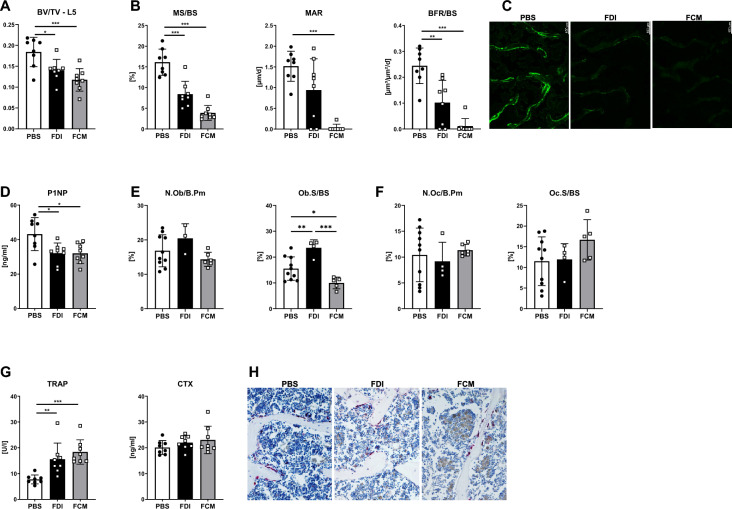
Histological and serological analysis of bone turnover. **A** Fraction of bone volume over total volume (BV/TV) was quantified using histological slides of the fifth lumbar vertebrae. **B, C** Dynamic histomorphometry of the fourth lumbar vertebrae indicates the mineralizing surface per bone surface (MS/BS), mineral apposition rate (MAR), and the bone formation rate per bone surface (BFR/BS). Representative images of calcein staining are shown in **C,** scale bar: 100 µm. **D** Serum procollagen type I N-terminal peptide (P1NP) levels were measured via ELISA. **E, F** Number per bone perimeter and surface area of osteoblasts and osteoclasts (N.Ob/B.Pm, Ob.S/BS, N.Oc/B.Pm, Oc.S/BS, respectively) were quantified at the fifth lumbar vertebrae. **G** Serum was collected to measure levels of tartrate-resistant acid phosphatase (TRAP) and C-terminal telopeptide of type I collagen (CTX). **H** Representative images of TRAP-stained vertebral sections, scale bar: 100 µm. Individual dots represent individual mice. Mean and SD are indicated as horizontal lines. Eight mice per group were used. A one-way ANOVA was used for statistical analysis. *p < 0.05, **p < 0.01, ***p < 0.001

To address how FDI and FCM affect bone turnover, we performed dynamic bone histomorphometry and analyzed serum bone turnover markers. At histological level, both FDI and FCM led to a reduced bone volume (Fig. 5A), supporting our hypothesis that the µCT picked up false-positive signals from the bone marrow in the FCM group resulting in unaltered bone volume. Both iron formulations led to a drastic reduction in mineralized surface, mineral apposition rate, and the bone formation rate with almost no calcein labels seen in the iron-treated groups (Fig. 5B, C). Accordingly, serum levels of the bone formation marker P1NP were reduced by 25% in both groups (Fig. 5D). FDI treatment increased both osteoblast number per bone perimeter and surface area, yet this expansion proved insufficient to restore normal bone mineralization (Fig. 5E). The FCM group exhibited the opposite pattern, with diminished osteoblast numbers. No significant changes were found in osteoclast number and osteoclast surface (Fig. 5F, H). Serum TRAP levels were increased with FCM and FDI, while serum CTX levels were not different between the groups (Fig. 5G). Despite no changes in bone volume, ID treatment also led to an inhibition of bone formation as displayed by the reduced levels of P1NP with no alterations of serum TRAP or CTX levels (Suppl. Figure 1I–K). Taken together, all iron formulations drastically decreased the bone formation rate with smaller effects on osteoclasts.

### FDI and FCM result in increased osteoid production and high FGF-23 levels

**Fig. 6:**
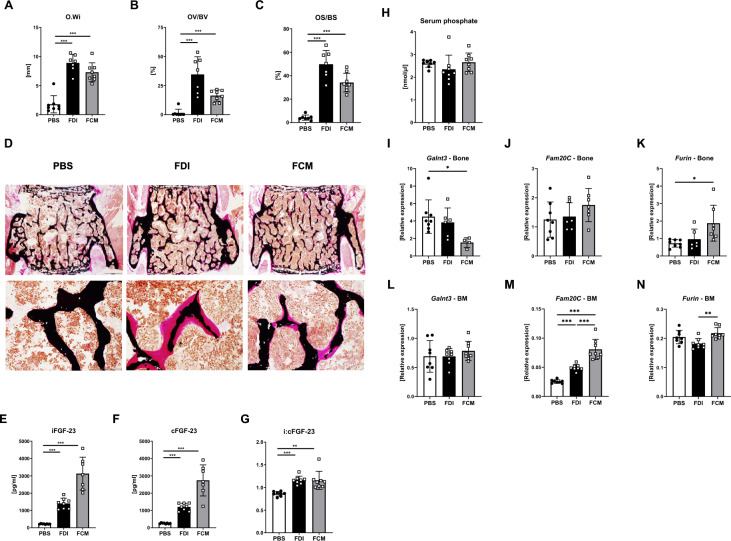
Increase in osteoid and serum levels of FGF-23 in mice treated with FDI and FCM. **A** Osteoid width (O.Wi), **B** osteoid volume/bone volume (OV/BV), and **C** osteoid surface/bone surface (OS/BS) were measured at the fourth vertebral body on undecalcified sections. Representative images of osteoid (pink) are shown in **D**. Upper panel scale bar: 500 µm. Lower panel scale bar: 100 µm. Serum levels of **E** phosphate, **F** intact FGF-23 and **G** C-terminal FGF-23 measured via ELISAs and used to calculate **H** the ratio of intact to C-terminal FGF-23. Expression of *Galnt3*, *Fam20C* and *Furin* in **I**-**K** bone and **L**–**N** bone marrow was measured independently by qRT-PCR. Individual dots represent individual mice. Mean and SD are indicated as horizontal lines. Eight mice per group were used. A one-way ANOVA was used for statistical analysis. *p < 0.05, **p < 0.01, ***p < 0.001

As the administration of FCM has been associated with transient hypophosphatemia, an increase in FGF-23 levels and potentially osteomalacia, we analyzed osteoid, serum phosphate and FGF-23 levels as well. FDI and FCM led to a marked increase in osteoid width, osteoid volume, and osteoid surface compared to PBS-treated mice (Fig. 6A–C). Large osteoid seams were observed in the iron-treated mice, especially in mice treated with FDI (Fig. 6D). Both FDI and FCM treatment resulted in increased intact and C-terminal serum levels of FGF-23, with FCM leading to higher increases than FDI (Fig. 6E, F). As the intact FGF-23 was more strongly up-regulated (sixfold with FDI and 13.5-fold with FCM) than the C-terminal FGF-23 (5.7-fold with FDI and 12.9-fold with FCM), the i:cFGF-23 ratio increased in both FCM- and FDI-treated mice (Fig. 6G). Paralleling observations in males, FCM and FDI upregulated intact FGF-23 levels in females (twofold with either FDI or FCM), although to a lower extent; cFGF-23 levels, by contrast, remained unchanged (Suppl. Figure 2N-P). However, no significant changes were observed in serum phosphate levels among treatments in males or females (Fig. 6H, Suppl. Figure 2 M). ID treatment elicited similar response patterns in serum phosphate, iFGF-23 and cFGF-23 levels (Suppl. Figure 1L, M). The magnitude of FGF-23 upregulation, however, was more modest, compared to that in FCM and FDI treatments, with no corresponding alterations in the i:cFGF-23 ratio (Suppl. Figure 1N).

Given that osteocytes are the main regulators of FGF-23, we examined expression of FGF-23 regulatory genes in bone tissue. FCM led to a downregulation of *Galnt3* and an upregulation of *Furin* in the bone (Fig. 6I-K). Although baseline transcription of these genes in the bone marrow was minimal, both FCM and FDI significantly upregulated *Fam20C* (Fig. 6L–N).

### A single injection of FDI and FCM results in milder effects on bone than repeated injections

Even though mouse studies frequently use repeated doses of iron to assess its effects on bone, we wanted to better mimic the clinical application of iron and thus, administered FDI and FCM only once and analyzed the bone outcomes after four weeks. Despite significant iron overload in several tissues (liver, spleen, bone marrow, bone), FDI and FCM treatment did not affect the body weight at the end of the experiment (Suppl. Table 1). Within the blood, FDI only increased white blood cell counts and reduced the number of reticulocytes (Suppl. Table 1). FCM in contrast already exerted stronger effects on the red blood cell compartment (reduced hematocrit, hemoglobin, MCV, reticulocytes) and elicited a stronger inflammatory response (higher numbers of white blood cells, in particular neutrophils) (Suppl. Table 1).

**Fig. 7:**
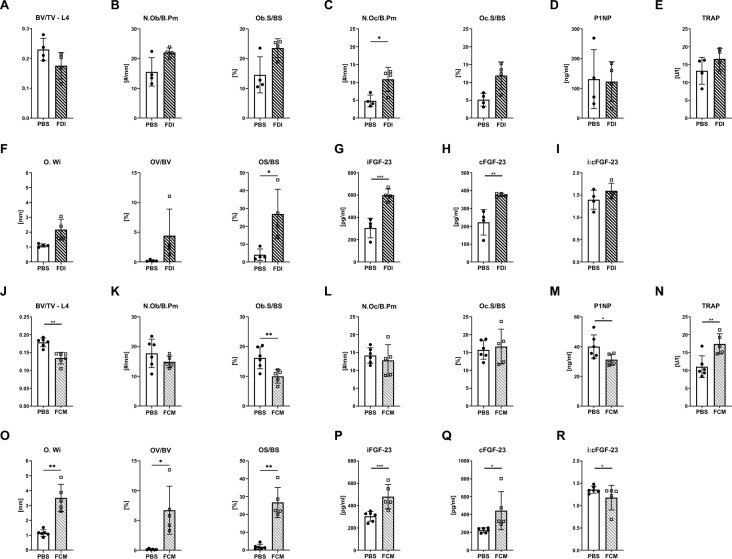
Abated effects from a single injection of FDI and FCM on osteoid formation and serum levels of FGF-23. Mice were treated with a single injection of FDI (N = 4 per group) **A-G** or FCM (N = 6 per group) **H-N**. Analyses were performed 4 weeks later. **A, J** Bone volume fraction (BV/TV) of the fourth lumbar vertebrae analyzed with µCT. **B, C, K, L** Number per bone perimeter and surface area of osteoblasts and osteoclasts (N.Ob/B.Pm, Ob.S/BS, N.Oc/B.Pm, Oc.S/BS, respectively) were quantified by histomorphometry at the fifth lumbar vertebrae. **D, E, M, N** P1NP and TRAP5b were measured from collected serum. **F, O** Osteoid width (O.Wi), osteoid volume/bone volume (OV/BV), and osteoid surface/bone surface (OS/BS) were assessed at the fourth lumbar vertebra. Serum levels of **G, P** intact FGF-23 and **H, Q** C-terminal FGF-23 were measured via ELISAs and used to evaluate **I, R** the ratio of intact to C-terminal FGF-23. Individual dots represent individual mice. Mean and SD are indicated as horizontal lines. A two-sided *t*-test was used for statistical analysis. *p < 0.05, **p < 0.01, ***p < 0.001

In the bone, FDI led to mild reduction of bone volume due to increased osteoclast numbers (Fig. 7A–C). Accordingly, serum TRAP levels were non-significantly increased by 25%, while serum levels of P1NP were not changed (Fig. 7D, E). Nonetheless, FDI already increased osteoid surface per bone surface sixfold as well as intact and C-terminal levels of FGF-23, which however did not lead to an increase in the i:cFGF-23 ratio (Fig. 7F–I). In contrast, even after a single injection of FCM, bone volume was decreased with a significant diminution of osteoblast surface (Fig. 7J–L). Consistently, serum levels of P1NP were significantly reduced, while TRAP levels were increased by 58% four weeks after a single FCM injection (Fig. 7M, N). FCM increased osteoid surface 14-fold along with osteoid width (threefold) and osteoid volume (sevenfold) with just one dose (Fig. 7O). Similar to FDI, FCM increased intact and C-terminal FGF-23; however, did not result in an increased ratio (Fig. 7P–R).

## Discussion

Intravenous administration of FDI and FCM are two of the most broadly applied therapeutic interventions for iron-deficiency anemia. Compared to the oral form as well as previous dextran-based formulations, they present outstanding features including higher tolerability, higher stability in the bloodstream leading to a superior ability to correct iron-deficiency anemia. These benefits are owed to their iron-oxyhydroxide structure, which has been shown to be closer to akaganeite rather than magnetite (Blumenstein et al. 2021). As a result, their dissolution rate is lower, rendering them not only more available in the circulation but also present for a longer time. The formulations therefore make it possible to give patients a large amount of iron in a single dose with less toxicity.

Despite the benefits mentioned above, the occurrence of transient hypophosphatemia has been widely reported after injection of FCM (Wolf et al. 2020a; Blumenstein et al. 2021; Boots and Quax 2022; Shahani et al. 2023). Hypophosphatemia is driven by increased serum levels of FGF-23, which is associated with reduced levels of the active form of vitamin D and calcium, followed by increased levels of parathyroid hormone (PTH) (Wolf et al. 2013, 2018, 2020a). Hypophosphatemia caused by administration of FCM was not only reported more frequently than with FDI, but also more severe (Wolf et al. 2020a, b). Even though FCM-induced hypophosphatemia is transient in most cases, case studies have shown that repeated administration of FCM may expose patients to a higher risk of developing osteomalacia, which is associated with bone pain and poor bone strength (Sangrós Sahún et al. 2016; Schaefer et al. 2017; Bartko et al. 2018; Klein et al. 2018; Wolf et al. 2018, 2020b; Fang et al. 2019; Tozzi and Tozzi 2020; Amarnani et al. 2020; Callejas-Moraga et al. 2020; Vilaca et al. 2022; Boots and Quax 2022). However, so far, no prospective studies have been performed to address the effect of long-term high dosing of FCM on bone mineralization. Thus, our study served to examine the effects of accumulating high doses of FCM and FDI on bone mineralization.

To investigate the direct effects of FDI and FCM on bone health in more detail, we used healthy male and female mice as a model to exclude confounders from other underlying diseases that induce anemia and at the same time may affect bone turnover such as inflammatory bowel disease or chronic kidney disease. In addition, to further bridge our mouse model findings to clinical use, we accounted for species-specific differences in iron handling and tolerance. In humans, both dietary iron absorption and iron excretion are tightly regulated. Critically, humans lack active mechanisms to excrete excess iron. Men have no physiological route for iron loss, while women can lose iron through menstruation. On the other hand, other mammals such as mice were reported to possess tolerance to very high doses of iron, up to several grams per kilogram (Whittaker et al. 1997; Huang et al. 2013; Xiong et al. 2022). For example, the C57BL/6 mouse strain showed cardio protection even with weekly intraperitoneal injections of 1.0 g/kg iron dextran for 8 consecutive weeks (Musumeci et al. 2013). Mechanistically, the existence of an excretory process via feces was shown to explain the tolerance in mice (Musumeci and Maccari 2014; La Carpia et al. 2019; Francis et al. 2019). Also, different rates of physiological and drug metabolism need to be considered in mice and humans, which were accounted for in this study. Thus, with these insights, we have mimicked a scenario of high iron dosing in healthy mice to assess the effects of iron overload on bone without confounders influencing the results.

As expected, we observed accumulation of iron in the liver and serum in mice that received parental iron. Transferrin in the circulation was nearly completely saturated with iron. Repeated dosing of FDI and FCM significantly reduced body weight after the 4-week observation period. In addition to the iron overload, mice developed a hyperchromic microcytic anemia, likely due to exhaustion of erythropoiesis, together with an increased number of white blood cells, which may indicate an inflammatory reaction to this high dose of iron. In other studies, iron overload by repeated infusion of either iron dextran, FCM or FDI was also reported to impair the hematopoietic system, while elevating white blood cell counts across various animal models, regardless of baseline iron status (Chai et al. 2015; Dominguez Rieg et al. 2025). Importantly, in our study, a single dose of iron also resulted in iron overload without showing an increase in white blood cell numbers, indicating that the repeated dosing led to iron intoxication of the mice, which goes along with inflammation and the formation of ROS.

Besides the systemic iron accumulation, we observed a significant accumulation of iron in the bone marrow compartment. In contrast to FDI-treated samples, FCM treatment resulted in iron-overloaded cells forming clusters rather than remaining as isolated individual cells, raising questions about the differences between the iron formulations as well as the cellular composition of these aggregates. Given their well-established role in iron handling and immune regulation (Knutson et al. 2005; Han et al. 2025), macrophages likely absorb the excess amount of iron from both formulations. Although the mechanisms by which FCM, but not FDI, induces cluster formation remain to be elucidated, previous studies have attributed the distinct clinical outcomes of FCM to its unique carbohydrate shell, which may influence macrophage behavior in our study. Based on this and established patterns of macrophage cooperation, it is plausible that these macrophage aggregates function cooperatively to clear iron-induced damaged cells, analogous to macrophage cooperation observed in other tissue injury contexts (Ohno et al. 2004; Zindel et al. 2021; Bill et al. 2023; Peiseler et al. 2023; Anstee et al. 2023; Look et al. 2025; Dooling et al. 2025). Peripheral neutrophil expansion suggests tissue recruitment and swarming. Given the degree of iron overload induced, inadequate macrophage-mediated clearance of neutrophil swarms (Uderhardt et al. 2019; Kraus and Gruber 2021) may explain elevated leukocyte counts and iron-loaded cell clusters in FCM-treated bone tissues. Specific signaling pathways and cellular identities require immunohistochemical characterization. On another note, a study showed that even though FCM is rapidly cleared in the serum after administration, it accumulates in multiple organs of mice, including heart, spleen, liver, for a long period after the infusion, up to 42 days (Vera-Aviles et al. 2024). Interestingly, similar myocardial iron accumulation was also observed in iron-deficient patients even after 365 days (Piechnik et al. 2025). These insights raise concerns about the possibility of iron accumulation in organs independent of the conventional safeguarding thresholds of iron overload such as ferritin and Tsat levels. Therefore, our current observation of iron depositions in the bone marrow offers a starting point for investigating the underlying mechanisms of the sustained iron accumulation in organs.

Accompanying iron accumulation in the bone marrow, iron-treated mice exhibited negative changes in bone parameters. The deleterious effect of iron on bone was even more prominent in those mice that received FCM compared to FDI. Both formulations remarkably reduced cortical thickness at the femoral mid-shaft. Moreover, tissue mineral density at the femur and L4 vertebra was also reduced by iron. Interestingly, loss of trabecular bone volume in the femur and L4 as assessed using µCT was only observed in FDI-treated mice. However, closer analyses using histology revealed that FCM produced a similar extent of trabecular bone loss, but these alterations were disguised in the µCT analyses by the formation of iron clusters in the bone marrow of FCM-treated mice that produced a false-positive signal. Due to these changes in the bone microarchitecture, FDI further reduced flexural strength assessed by the three-point bending test. Interestingly, there was no difference in mice treated with FCM compared to those receiving PBS. This resistance could be attributable to the distinct pattern of FCM deposition in the bone marrow.

The bone loss caused by the parenteral iron was mainly a result of defective mineralization by osteoblasts, rather than increased resorption. The persistently high levels of iron, whether provided via FDI or FCM, severely reduced all measured mineralization indices as well as serum P1NP level and accumulated unmineralized osteoid despite differential changes in osteoblast quantity. FDI increased osteoblast numbers, while FCM decreased them, indicating preserved osteoblast presence but impaired function with FDI and a more severe cytotoxic effect by FCM. The proposed cause of iron-induced bone loss was further supported by unchanged osteoclast parameters including the number of osteoclasts and serum CTX levels. As macrophages also express TRAP (Räisänen et al. 2005; How et al. 2014), it is possible that the iron-loaded macrophages in the bone marrow contributed to the elevated serum TRAP levels. These results can be supported by the fact that CTX is normal after iron treatments in our study. In contrast, the persistently high levels of iron, whether provided via FDI or FCM, severely reduced the number of osteoblasts and impaired their bone-forming properties. Previous studies have shown that osteoblasts are very sensitive to iron-induced ROS formation and ferroptosis. Blocking either iron-induced oxidative stress using N-acetyl cysteine (Tsay et al. 2010) or ferroptosis using ferrostatin-1 (Jiang et al. 2022) rescued bone formation in vitro and in vivo. Thus, our data strongly suggest that to maintain osteoblast function, iron concentrations should be kept to a minimum.

In contrast to studies in humans (Wolf et al. 2020a; Kassianides and Bhandari 2024), in our mouse model, both FDI and FCM induced serum levels of FGF-23. FCM led to a higher increase in intact and C-terminal FGF-23 than FDI, but both resulted in a higher i:cFGF-23 ratio owing to the higher magnitude of iFGF-23 production. It is plausible to believe that the physiological FGF-23 cleaving capacity of the body, to maintain a normal level of iFGF-23, is challenged by the high dose of these two intravenous iron formulations. Indeed, unlike the effect caused by the repeated doses, single dose of either FDI or FCM elevated both iFGF-23 and cFGF-23 levels but the ratio of i:cFGF-23 was not significantly increased.

While ectopic expression of FGF-23 in non-osseous cell types across various mouse models (Ishii et al. 2021; Li et al. 2023) has emerged in recent reports, bone tissues remain recognized as the main regulatory site of the growth factor in our study. This was supported by the higher expression of *Galnt3*, *Fam20C* and *Furin* in bone tissues versus bone marrow, indicating bone as the key FGF-23 processing hub. The downregulation of the iFGF-23-preserving *Galnt3* and the upregulation of cleavage-promoting factors such as *Fam20C* and *Furin* in bone likely reflect compensatory responses to iron-induced overwhelm of FGF-23 cleavage machinery. Increased c-terminal FGF-23 by osteocytes has been observed in inflammatory contexts (Courbon et al. 2023), suggesting that inflammation may also contribute to the bone loss in our study. Furthermore, in myelodysplastic syndromes, characterized by impaired hematopoiesis and skeletal abnormalities, elevated C-terminal FGF-23 correlates positively with non-mineralized bone accumulation and negatively with hemoglobin levels (Weidner et al. 2020), paralleling our observations. Despite the well-established role of intact FGF-23 in regulating serum phosphate and calciotropic hormones (vitamin D, PTH), we are not the first to observe uncoupling of elevated FGF-23 production from these canonical functions due to enhanced proteolysis. The mechanisms driving this enhanced cleavage warrant further investigation (Wang et al. 2008; Sitara et al. 2008; Weidner et al. 2020).

Regardless of the amount of i:cFGF-23, both formations induced a massive amount of osteoid. This may be the result of iron incorporating into the nascent bone matrix instead of calcium, which blocks further mineralization. Indeed, iron was reported to inhibit crystal growth of hydroxyapatite (Guggenbuhl et al. 2008), the most abundant component in bones. Similar findings were observed in rat and porcine models of osteomalacia induced by aluminum (Sedman et al. 1987; Rodriguez et al. 1990). These alterations support our hypothesis that the overdose of iron led to inflammation in the bone marrow and disrupted mineralization in the bone tissue. Although our qBEI data obtained from a deep layer inside the mineralized bone reflected no changes in calcium distribution, it does not exclude the possibility that iron impaired mineralization by competing with calcium.

Given well-documented skeletal sexual dimorphism in C57BL/6 J mice across multiple anatomical sites (lumbar vertebrae, femora, tibiae) and in different physiological (such as aging) as well as pathological conditions (Yao et al. 2013; Kerschan-Schindl et al. 2022; Welhaven et al. 2022; Jain et al. 2025), we conducted similar experiments in female mice to assess whether the aforementioned findings extend across sexes. Both FDI and FCM induced bone loss; however, the magnitude was attenuated in females compared to males. Nevertheless, the detrimental effects of iron overload on bone were significant in both sexes. Both FCM and FDI upregulated intact FGF-23 levels in females as in males; cFGF-23 remained unchanged in females, suggesting sex-specific FGF-23 processing differences. These patterns may reflect inherent sex differences in bone metabolism (Welhaven et al. 2022), warranting investigation into whether differential responses stem from variations in iron metabolism, FGF-23 signaling, or bone remodeling dynamics.

Despite careful planning and rigorous experimental design, our study has limitations. First, we studied the effects of FCM and FDI on the bones of healthy mice instead of using an iron-deficient or anemia model. However, this was a deliberate choice to exclude confounding factors that may further influence the regulation of FGF-23. In addition, we applied doses of iron higher than those used in humans for multiple reasons (considering the higher metabolic rate of mice, the dose is about twofold higher). This was done to simulate the long-term accumulation of iron, leading to high but not lethal doses of iron, which served as a proof-of-principle to study the effects on bone homeostasis, mineralization, and FGF-23 levels. The application of high doses also manifested the ability of healthy bodies to handle iron overload. Even though this protocol induced iron toxicity, animals receiving a single dose of FDI or FCM expressed similar, but less pronounced effects, suggesting that even lower doses of iron can induce FGF-23 levels and the formation of excess osteoid. Nevertheless, extrapolation to clinical iron exposure requires caution, as systemic iron intoxication in our model may contribute to bone loss through toxicity-related mechanisms. Lastly, due to technical limitations in immunostaining of bone sections, further methods are required to determine the contribution of osteocytes versus marrow cells to FGF-23 production.

In summary, our data reveal that high doses of both FDI and FCM negatively affected bone microarchitecture. Reduced bone mineralization and a high accumulation of osteoid associated with high levels of FGF-23 were the main underlying mechanisms. Between the two iron formulations, the negative effects on bone were more pronounced in mice treated with FCM, and both formulations showed dose-dependent effects.

## Supplementary Information

Below is the link to the electronic supplementary material.

Supplementary file1 (DOCX 19 KB)

Supplementary file2 (DOCX 19 KB)

Supplementary file3 (DOCX 117 KB)

## Data Availability

No datasets were generated or analysed during the current study.
