# Supplementary material for: Iron overload induced by ferric derisomaltose and ferric carboxymaltose both increase FGF-23 levels and lead to osteomalacia and bone loss in normal mice
Source: Biometals. 2026 Feb 13;39(3):953–71. doi: 10.1007/s10534-026-00794-x (PMC13230282; doi:10.1007/s10534-026-00794-x)
Supplement: Supplementary file 1 — Supplementary file1 (DOCX 19 KB) [file 10534_2026_794_MOESM1_ESM.docx]

|  | **Control**  N=4 | **FDI 1x**  N=4 | **Control**  N=8 | **FCM 1x**  N=8 |
| --- | --- | --- | --- | --- |
| Weight [g] | 30.3 ± 1.71 | 31.4 ± 1.61 | 27.9 ± 1.91 | 27.9 ± 0.49 |
| Red blood cells [10^6^/ µl] | 8.70 ± 0.31 | 8.47 ± 0.57 | 9.16 ± 0.77 | 8.29 ± 0.43 |
| Hematocrit [%] | 41.7 ± 1.40 | 40.7 ± 3.00 | 45.4 ± 4.20 | 39.2 ± 1.70* |
| Hemoglobin [g/dl] | 7.80 ± 0.25 | 7.73 ± 0.51 | 8.35 ± 0.67 | 7.50 ± 0.37* |
| MCV [fl] | 47.9 ± 0.44 | 48.1 ± 0.84 | 49.5 ± 0.67 | 47.4 ± 0.98** |
| MCH [pg] | 0.90 ± 0.02 | 0.91 ± 0.01 | 0.91 ± 0.02 | 0.91 ± 0.01 |
| MCHC [g/dl] | 18.7 ± 0.32 | 19.0 ± 0.17 | 18.4 ± 0.55 | 19.1 ± 0.42* |
| Platelets [10^3^/ µl] | 701 ± 139 | 644 ± 126 | 555 ± 147 | 563 ± 132 |
| White blood cells [10^3^/ µl] | 9.31 ± 0.83 | 12.04 ± 1.47* | 8.26 ± 2.04 | 14.2 ± 3.86** |
| Neutrophils [%] | 0.20 ± 0.06 | 0.34 ± 0.15 | 0.27 ± 0.13 | 0.62 ± 0.12** |
| Lymphocytes [%] | 2.84 ± 0.42 | 3.04 ± 0.42 | 2.31 ± 0.60 | 3.65 ± 0.91* |
| Monocytes [%] | 0.15 ± 0.15 | 0.37 ± 0.14 | 0.19 ± 0.14 | 0.43 ± 0.37 |
| Reticulocytes [10^9^/L] | 198 ± 19.5 | 136 ± 3.8** | 232 ± 34.3 | 157 ± 37.1** |
| Liver iron content [µg/g tissue] | 68.4 ± 54.6 | 6711.7 ± 3139.3*** | 107.7 ± 86.3 | 18126.1 ± 7964.6*** |

Supplementary Table 1. Blood counts in mice treated with a single dose of iron.

MCV = mean corpuscular volume. MCH = mean corpuscular hemoglobin. MCHC = mean corpuscular hemoglobin concentration. Data represent the mean ± SD. Statistical analysis was performed by the Student´s *t*-test. *p<0.05; **p<0.01; ***p<0.001.
