# Supplementary material for: Iron overload induced by ferric derisomaltose and ferric carboxymaltose both increase FGF-23 levels and lead to osteomalacia and bone loss in normal mice
Source: Biometals. 2026 Feb 13;39(3):953–71. doi: 10.1007/s10534-026-00794-x (PMC13230282; doi:10.1007/s10534-026-00794-x)
Supplement: Supplementary file 2 — Supplementary file2 (DOCX 19 KB) [file 10534_2026_794_MOESM2_ESM.docx]

Supplementary table 2. Blood counts in female mice treated with multiple doses of iron.

|  | **Control**  N=5 | **FDI**  N=5 | **FCM**  N=5 |
| --- | --- | --- | --- |
| Red blood cells [10^6^/ µl] | 9.16 ± 0.8 | 8.57 ± 1.41 | 9.4 ± 0.92 |
| Hematocrit [%] | 0.44 ± 0.04 | 0.41 ± 0.06 | 0.45 ± 0.04 |
| Hemoglobin [g/dl] | 8.46 ± 0.57 | 8.16 ± 1.44 | 8.7 ± 0.84 |
| MCV [fl] | 48.54 ± 0.51 | 48.84 ± 2.01 | 48.28 ± 0.87 |
| MCH [pg] | 0.92 ± 0.02 | 0.94 ± 0.02 | 0.92 ± 0.01 |
| MCHC [g/dl] | 19.06 ± 0.64 | 19.46 ± 0.65 | 19.18 ± 0.51 |
| Platelets [10^3^/ µl] | 631.2 ± 350.41 | 481.8 ± 181.12 | 530.4 ± 69.07 |
| White blood cells [10^3^/µl] | 8.97 ± 4.88 | 10.95 ± 3.68 | 10.24 ± 2.41 |
| Neutrophils [%] | 6.62 ± 4.49 | 9.6 ± 3.96 | 10.02 ± 2.45 |
| Lymphocytes [%] | 82.66 ± 13.3 | 83.34 ± 7.49 | 73.56 ± 10.17 |
| Monocytes [%] | 9.86 ± 12.54 | 6.4 ± 3.16 | 16.02 ± 11.79 |
| Reticulocytes [10^9^/L] | 277.68 ± 69.66 | 134.04 ± 45.59****** | 159.72 ± 41.82***** |

MCV = mean corpuscular volume. MCH = mean corpuscular hemoglobin. MCHC = mean corpuscular hemoglobin concentration. Data represent the mean ± SD. Statistical analysis was conducted using the Student´s *t*-test for comparisons between Control and Iron dextran, and one-way analysis of variance (ANOVA) followed by Tukey’s post-hoc test for comparisons among Control, FDI and FCM. *p<0.05; **p<0.01; ***p<0.001.
