# Supplementary material for: Iron overload induced by ferric derisomaltose and ferric carboxymaltose both increase FGF-23 levels and lead to osteomalacia and bone loss in normal mice
Source: Biometals. 2026 Feb 13;39(3):953–71. doi: 10.1007/s10534-026-00794-x (PMC13230282; doi:10.1007/s10534-026-00794-x)
Supplement: Supplementary file 3 — Supplementary file3 (DOCX 117 KB) [file 10534_2026_794_MOESM3_ESM.docx]

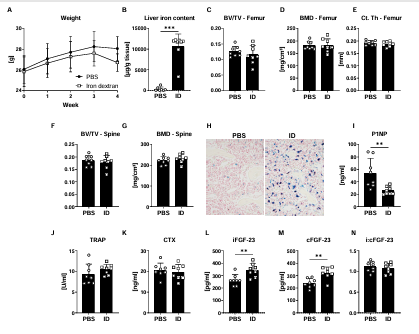


Supplementary figure 1

**Supplementary Figure 1. Effects of ID on iron overload, marrow iron, bone microarchitecture and bone turnover.**

Mice were injected with ID once per week for four weeks (N=8 per group). Dosage is similar to the one used in FDI and FCM experiments. **(A)** The body weight of the animals was followed over the course of the experiment. **(B, H)** Iron accumulation was assessed in the liver and the bone marrow via the measurement of liver iron content level and histology, respectively. **(C-G)** Bone volume fraction (BV/TV), bone mineral density (BMD) and cortical thickness (Ct.Th) of the femur and vertebra were quantified using µCT. **(I-K)** Serum levels of P1NP, TRAP and CTX reflected bone remodeling activity after the ID treatment. Serum levels of **(L)** intact and **(M)** C-terminal FGF-23 were measured via ELISAs and used to evaluate **(N)** the ratio of intact to C-terminal FGF-23. Individual dots represent individual mice. Mean and SD are indicated as horizontal lines. A two-sided *t*-test was used for statistical analysis. *p<0.05, **p<0.01, ***p<0.001.


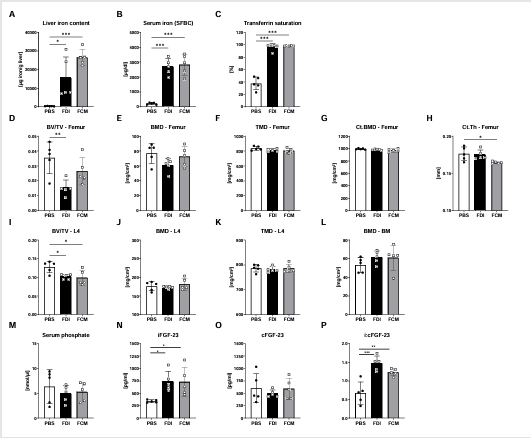


Supplementary figure 2.

**Supplementary Figure 2. Bone loss and upregulation of intact FGF-23 induced by FCM and FDI**

Similar treatments of FCM and FDI were applied to age-matched female mice (N=5 per group). Iron overload was assessed in the **(A)** liver and **(B, C)** serum. **(D, I)** Bone volume fraction (BV/TV), **(E, J)** trabecular bone mineral density (BMD), **(F, K)** tissue mineral density (TMD), **(G)** cortical bone mineral density (Ct. BMD) cortical thickness (Ct.Th) of the femur and vertebra as well as **(L)** bone mineral density of the bone marrow compartment were quantified using µCT. **(I-K)** Serum levels of P1NP, TRAP and CTX reflected bone remodeling activity after the ID treatment. Serum levels of **(M)** phosphate, **(N)** intact and **(O)** C-terminal FGF-23 were measured via ELISAs and used to evaluate **(P)** the ratio of intact to C-terminal FGF-23. Individual dots represent individual mice. Mean and SD are indicated as horizontal lines. A one-way ANOVA was used for statistical analysis. *p<0.05, **p<0.01, ***p<0.001.
